# IgE Mediated Autoallergy against Thyroid Peroxidase – A Novel Pathomechanism of Chronic Spontaneous Urticaria?

**DOI:** 10.1371/journal.pone.0014794

**Published:** 2011-04-12

**Authors:** Sabine Altrichter, Hans-Jürgen Peter, Dina Pisarevskaja, Martin Metz, Peter Martus, Marcus Maurer

**Affiliations:** 1 Department of Dermatology and Allergy, Allergie-Centrum-Charité/ECARF, Charité - Universitätsmedizin Berlin, Berlin, Germany; 2 Department of Biometry and Clinical Epidemiology, Charité - Universitätsmedizin Berlin, Berlin, Germany; The University of Queensland, Australia

## Abstract

**Background:**

Chronic spontaneous urticaria (csU), which is characterized by recurrent episodes of mast cell-driven wheal and flare-type skin reactions, is often associated with elevated total IgE levels and thyroid autoimmunity. We speculate that some csU patients express IgE autoantibodies against thyroid antigens such as thyroid peroxidase (TPO), which could bind to skin mast cells and induce their activation.

**Methods:**

We developed and used a site-directed human IgE capture ELISA to quantify IgE-anti-TPO. We used this assay and investigated csU patients (n = 478) and healthy control subjects (n = 127) for IgE-anti-TPO and then assessed IgE-anti-TPO-positive and -negative csU patients for clinical and serological differences.

**Principal Findings:**

CsU patients were found to express more than 2fold higher IgE-anti-TPO serum levels as compared to healthy control subjects (p<0.001). 54% of csU patients had serum levels higher than the cut off ( = 5 IU/ml). By distribution analyses we identified two distinct subpopulations of csU patients: 1) IgE-anti-TPO^low^ ( = 39%, IgE-anti-TPO: median 2.17 interquartile range 0.86–5.44, =  comparable to healthy controls) and 2) IgE-anti-TPO^high^ ( = 61%, IgE-anti-TPO: median 6.67, interquartile range 5.39–8.24). IgE-anti-TPO-positive and -negative csU patients had very similar distributions of age and gender as well as disease activity and duration. IgE-anti-TPO-positive csU patients exhibited significantly higher IgG-anti-TPO levels and lymphocyte counts as well as decreased C4 complement levels.

**Conclusion:**

Our findings show that a sizeable subgroup of csU patients expresses IgE antibodies against thyroid peroxidase. These autoantibodies could cause “autoallergic” mast cell activation, a novel pathomechanism of chronic spontaneous urticaria.

## Introduction

Urticaria is a common condition characterized by itchy wheal and flare type skin reactions (hives) and/or angioedema [1]. These symptoms are brought about by activated skin mast cells and their subsequent release of histamine and other proinflammatory mediators [2]. The underlying causes and the mechanisms of mast cell activation in most types of urticaria are largely unknown and remain to be identified.

Based on clinical observations, several pathways of mast cell activation in urticaria have been proposed. For example, patients with chronic spontaneous urticaria (csU), the most frequent type of non acute urticaria, have repeatedly been described to exhibit increased levels of IgE. In a recent study, 50% of csU patients exhibited significantly (i.e. more than 4fold) elevated levels of total serum IgE (>100 IU/ml) as compared to only 13% of healthy control subjects [3]. This raises the possibility that in csU mast cells may be activated by allergens that engage specific IgE antibodies bound to their high affinity IgE receptor, Fc_epsilon_RI. However, sensitisations to aeroallergens or other environmental allergens, even uncommon ones, are rarely found to be the cause of csU [3],[4].

Also, csU patients have been reported to frequently suffer from autoimmune conditions, especially thyroid autoimmune disorders such as Hashimoto's thyroiditis [5],[6]. Several independent studies have demonstrated that a significant number of csU patients (in some studies up to 33%) exhibit high levels of autoantibodies to thyroid antigens [7]. As of now, a role of thyroid autoimmunity and thyroid antibodies in mast cell activation in csU remains to be proven.

Here, we postulate that skin mast cells in some csU patients are activated by an ‘autoallergic’ mechanism. Specifically, we speculate that patients with thyroid autoimmunity and IgG autoantibodies to thyroid antigens can also exhibit IgE autoantibodies to these autoantigens that then function as ‘autoallergens’. To test our hypothesis, we have developed an ELISA-based detection assay for IgE antibodies to thyroid peroxidase (TPO) and we have tested csU patients and healthy subjects for the presence of these IgE autoantibodies to TPO (IgE-anti-TPO).

## Results

### Chronic spontaneous urticaria patients exhibit elevated levels of IgE against thyroid peroxidase (IgE-anti-TPO)

**Figure 1:**
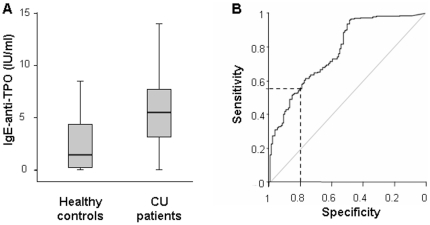
Patients with chronic spontaneous urticaria exhibit elevated levels of IgE-anti-TPO. Fig. 1a Box-Plot: Healthy persons (n = 127) exhibit in the mean IgE-anti-TPO levels of 2.58 IU/ml±2.46, 1st quartile 0.27– median 1.46– 3rd quartile 4.45 (median 1.46, IQR 0.27–4.45 IU/ml). Highest value among the healthy persons was 7.8 IU/ml. CU patients (n = 478) show elevated mean levels of 5.69 IU/ml±3.17, 1st quartile 3.2– median 5.5– 3rd quartile 7.73, (median 5.50, IQR 3.2–7.7 IU/ml), highest value 18.0 IU/ml. The differences between the two groups are statistical significant (p<0.001). Figure 1b ROC-Curve: Diagnostic accuracy for the distinction of CU patients and healthy controls was analysed by ROC curve including selected pairs of sensitivity and specificity (Table 1), the area under the curve (AUROC = 0.78) and the confidence limits for this curve (CI = 0.74–0.83). The selected specificity of 0.8 and the resulting sensitivity of 0.55 are displayed as dotted line in the graph.

Levels of IgE-anti-TPO, as assessed by site-directed IgE capture ELISA, were found to be significantly higher in csU patients (median 5.50, interquartile range IQR 3.2–7.7) as compared to healthy subjects (median 1.46, IQR 0.27–4.45 IU/ml) (Fig. 1a). Based on the ROC analysis of these findings (Fig. 1b) we defined 5.0 IU/ml as the cut off value (specificity = 0.8, Table 1) and found that 259 of 478 csU patients (54.2%) exhibit elevated levels of IgE-anti-TPO.

**Table 1:** Cut-off values, sensitivities and specificities for IgE-anti-TPO.

Cut-Off (IU/ml)	1.20	1.80	2.30	2.70	3.20	3.60	4.10	4.60	*5.00*	5.50	6.40
Sensitivity	0.95	0.90	0.85	0.80	0.75	0.70	0.65	0.60	*0.55*	0.50	0.42
Specificity	0.49	051	0.53	0.54	0.56	0.62	0.70	0.78	*0.80*	0.86	0.90

### CU patients are IgE-anti-TPO^low^ or IgE-anti-TPO^high^

**Figure 2:**
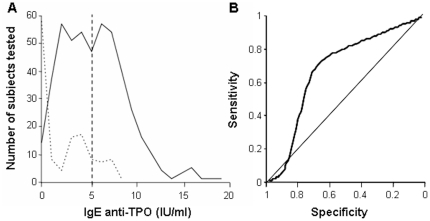
CU Patients can be divided into IgE-anti-TPO^high^ and IgE-anti-TPO^low^ subgroups. Figure 2a Density Estimation: CU patients (full line) and healthy controls (dotted line) were clearly different regarding their IgE-anti-TPO levels. CU patients can be subdivided in two subgroups, one similar to the healthy controls and the other one above the indicated cut off level of 5 IU/ml. Figure 2b Theoretical ROC-Curve: The theoretical ROC-Curve shows the result of the applied method of mixed distribution to identify subgroups within the patients' sample. This analysis was motivated by the hypothesis that only for a subtype of the disease IgE is involved in the pathological pathway. For the resulting normal distribution of the second subpopulation with elevated IgE-anti-TPO (IgE-anti-TPO^high^) values the theoretical ROC curve is displayed.

The distribution of IgE-anti-TPO levels in csU patients showed two distinct peaks, one very similar to that found in healthy controls and a second at above cut off IgE-anti-TPO levels (Fig. 2a). Indeed, distribution analyses of IgE-anti-TPO levels in csU patients showed two distinct subpopulations, i.e. the observed distribution of IgE-anti-TPO levels in csU patients resembled a mixture of two normal distributions with different means and different standard deviations (IgE-anti-TPO^low^ and IgE-anti-TPO^high^ patients). IgE-anti-TPO^low^ csU patients (39% of all csU patients) exhibited IgE-anti-TPO levels that were very similar to those of healthy controls (median 2.17 IQR 0.86–5.44 IU/ml). In contrast, the median level in IgE-anti-TPO^high^ patients (61% of all csU patients) was 6.67 (IQR 5.39–8.24 IU/ml (Fig. 2b). This two population model was highly significant as compared to a one component model (p<0.001). The theoretical ROC curve is presented in figure 2b.

### Levels of autoantibodies and lymphocytes are high and complement levels are low in csU patients with elevated IgE-anti-TPO

CsU patients with above cut off IgE-anti-TPO levels (IgE-anti-TPO+) were indistinguishable from those with below cut off levels (IgE-anti-TPO–) in terms of age, gender ratio, duration or severity of disease and total IgE serum levels. There also was no significant difference of the rates of ASST positive patients between the two patient populations, although the IgE-anti-TPO+ patients were largely ASST negative (Table 2). In contrast, IgE-anti-TPO+ patients showed significantly higher levels of IgG-anti-TPO as well as lymphocyte numbers and lower levels of complement C4 as compared to IgE-anti-TPO– csU patients (Table 3).

**Table 2:** Similarities and differences of IgE-anti-TPO+ and IgE-anti-TPO- CU patients.

	IgE-anti-TPO+	IgE-anti-TPO-	Statistical significance
**Female**	200 of 265 (75.5%)	166 of 213 (77.9%)	n.s.
**Age (years)**	45.5±14.5 (n = 265)	47.8±14.6 (n = 213)	n.s.
**Duration of disease (years)**	7.1±10.4 (n = 103)	5.9±9.7 (n = 50)	n.s.
**UAS**	2.41 (2.0 (n = 17)	2.50 (1.8 (n = 52)	n.s.
**DLQI (sum)**	7.8 (5.4 (n = 71)	8.9 (7.2 (n = 85)	n.s
**Total serum IgE (IU/ml)**	175,16±335,74 (n = 121)	221,10±332,07 (n = 213)	n.s.
**Positive ASST (% of n)**	16,7 (n = 30)	30,8 (n = 26)	n.s.

Values are given as mean ± standard deviation, exept for ASST (given in %).

**Table 3:** IgE-anti-TPO+ CU patients, but not IgE-anti-TPO- CU patients, show correlations of IgE-anti-TPO with IgG-anti-TPO, lymphocyte counts and complement C4 levels.

	IgE-anti-TPO+	IgE-anti-TPO-
**IgG-anti-TPO**	ρ** = 0.285 p = 0.002 (n = 113)**	ρ = 0.129 p = 0.327 (n = 60)
**Complement C4**	ρ** = −0.246 p = 0.006 (n = 113)**	ρ = −0.071 p = 0.552 (n = 72)
**Lymphocytes**	ρ** = 0.208 p = 0.019 (n = 127)**	ρ = −0.023 p = 853 (n = 70)

Values are given as Correlation Coefficient ρ (Spearman's Rank Test).

## Discussion

Here, we show for the first time that a sizeable subgroup of patients with chronic spontaneous urticaria exhibits IgE antibodies against self, i.e. against thyroid peroxidase. These IgE-anti-TPO autoantibodies, when bound and activated on the surface of mast cells, could cause ‘autoallergic’ mast cell degranulation, a novel pathogenic pathway of urticaria induction. We used the method of univariate mixture analysis to analyse the dependence between IgE-anti-TPO and CsU. This method is useful to detect and to explain heterogeneity in data. It should be mentioned, however, that the interpretation of mixture components as subpopulations is only one possible option.

Mast cell activation in csU has repeatedly been shown to involve autoantibodies, for example, IgG autoantibodies directed against IgE [8] or its high affinity receptor Fc_epsilon_RI [9]. These IgG autoantibodies can be detected in 24% to 60% of csU patients [10]–[12] and they have been shown to be relevant in patients with autoreactive csU, i.e. csU patients that are positive in the autologous serum skin test (ASST) [12], [13]. In contrast, IgE autoantibodies, such as those detected in about 50% of csU patients in our study, have not been described as relevant for the activation of mast cells in csU, except for in single patients. Ten years ago, Bar Sela and coworkers were able to detect such antibodies in the serum of a female patient who suffered from csU and Hashimoto's thyroiditis [14]. Subsequent studies using classic ELISA or RAST were unable to reproduce these findings in patients with csU and thyroid autoimmunity [15], probably due to interfering IgG autoantibodies [16] and the limited sensitivity of these assays. We were able to detect IgE specific for TPO by classical ELISA (Supplemental Figure S1b). Only after depleting competing IgG autoantibodies from the patient serum via protein G affinity chromatography and after removing all minor proteins by centrifugal ultrafiltration (Supplemental Figure S1c). In contrast, the new human-IgE-capturing-enzyme immunoassay developed and used in the present study allows for the detection of IgE-autoantibodies specific to TPO without prior depletion of IgG antibodies. (Supplemental Figure S1a)

Autoallergic mast cell activation has been implied to play a role in other chronic inflammatory skin disorders such as atopic dermatitis [17], [18] and bullous pemphigoid [19], [20], [21]. In both diseases IgE antibodies directed to skin antigens have been described and may play a role in the pathogenesis by activating cutaneous mast cells after binding their corresponding skin antigen. In contrast, the IgE autoantibodies detected in our study are directed against an extracutaneous antigen ( =  autoallergen), i.e. TPO, which can be released from the thyroid into the circulation. IgE-anti-TPO is, therefore, likely to be bound to mast cells, basophils and other Fc_epsilon_RI-expressing cells throughout the body. This may explain, why symptoms in csU patients are not limited to the skin, as in atopic dermatitis and bullous pemphigoid, but can also involve the gut, the airways, the joints and other organs.

For atopic dermatitis it was postulated that mimicry of protein domains of external allergens and self-proteins may turn an allergy against an environmental allergens into an autoallergy [22], [23], [24]. This is unlikely to be the case in csU, which is rarely associated with allergies to environmental allergens. However, autoallergen mimicry cannot be excluded in csU, as the extracellular domain of TPO has a similarity of around 45% with myeloperoxidases from eosinophils [25]. Also, IgE-anti-TPO production and detection may involve peroxidases of common cutaneous pathogens, including fungi.

CsU patients have been repeatedly reported to show a high incidence of autoimmune thyroiditis [6], [26], and up to 33% of patients reportedly express increased IgG antibodies directed against thyroid peroxidase or thyreoglobuline as compared to 5% in healthy individuals [7]. Also, csU patients exhibit significantly (more than 4fold) increased levels of total serum IgE (>100 U/ml) [3]. In contrast, relevant sensitizations against common environmental allergens are rarely found in csU patients. These findings may be explained, at least in part, by the results of our study: 1) IgE-anti-TPO expression is positively correlated to IgG-anti-TPO expression. In other words: IgG-anti-TPO-positive patients are more likely to express potentially urticaria-inducing IgE-anti-TPO. 2) IgE-anti-TPO may contribute to higher total IgE levels in csU patients.

With autoreactivity, intolerance, infection, and other underlying conditions shown to be relevant causes of csU, it is unlikely that IgE-anti-TPO is the relevant mast cell activator in all csU patients. Our statistical analyses using the method of mixed distribution identified 2 distinct subgroups – one IgE-anti-TPO^low^ and the other IgE-anti-TPO^high^. This supports the theory that autoallergy is of pathological relevance only in a subpopulation of csU patients. However, IgE autoantibodies directed against other autoantigens may exist and act as relevant mast cell activators in those patients shown to express low levels of or no IgE-anti-TPO [27].

Interestingly, IgG-anti-TPO and IgE-anti-TPO are positively correlated in IgE-anti-TPO+ but not IgE-anti-TPO- csU patients, suggesting that IgG-anti-TPO may be a good screening marker for the presence of IgE-anti-TPO and vice versa. Also, high IgE-anti-TPO is correlated with increased lymphocyte counts and C4 consumption, two common features of autoimmune conditions. There is growing consus that in a subgroup of patients csU should be regarded as an autoimmune disease with potential upregulation of previously unrecognized autoimmune manifestations. It needs to be proven that these manifestations are directly related and relevant to the pathogenesis of disease in the affected patient population.

Taken together, we can show that IgE-anti-TPO autoantibodies are common in csU patients. These findings point towards an autoallergic mechanism of mast cell activation as a novel and relevant pathogenetic mechanism in csU. Our results also encourage to screen csU patients for IgE directed against autoantigens other than TPO and to test IgE-neutralizing strategies such as omlizumab for their efficacy in csU.

## Materials and Methods

### Patients

Sera obtained from 478 consecutive csU patients treated at the Department of Dermatology, Charité – Universitätsmedizin Berlin were tested for IgE-anti-TPO levels (Table 4). As controls, sera from 127 age- and sex-matched healthy subjects were used (Table 4). All subjects gave written informed consent. This study was approved by the ethics committee of the Landesärztekammer Rheinland-Pfalz and done in accordance with the Declaration of Helsinki.

**Table 4:** Patient statistics.

	CU patients	Healthy controls
**Number**	478	127
**Female (%)**	72.4	76.6
**Age (years)** *	43.3±17.0	46.5±14.5

*Values are given as mean ± standard deviation. There are no significant differences between the two groups.

#### Detection of IgE-anti-TPO

IgE-anti-TPO serum levels were assessed by a site-directed IgE capture ELISA (depicted in Supplemental figure S1a). To this end, serum IgE was first captured using hydrazine surface plates (Costar Corning, Badhoevedoerp, Netherlands) coated with site-directed Fc epsilon-specific anti-human IgE antibodies from goat serum (Sigma-Aldrich, Deisenhofen, Germany). After blocking with 10% FCS in PBS, plates were incubated consecutively with serum (diluted 1∶25, for 120 minutes, at 20°C), followed by biotinylated (biotin XX-labeling kit, Pierce, Rockford, IL, USA) recombinant human-TPO (RSR Ltd, Cardiff, UK), streptavidine alkaline phosphatase (1∶10,000; Jackson Immunoresearch, West Grove, PA, USA), and its substrate p-NPP (Sigma-Aldrich, Deisenhofen, Germany). Between each step intensive washing with TBS containing 0.025% Tween 20 was performed. As a last step, the enzymatic dye reaction was stopped with 3 M NaOH and the optical density was measured at 405 nm using a MultiScan Ascent plate reader. Chimaeric human IgE-anti-TPO was obtained from the supernatant of SP-2/Sp1,4 transfected mouse myeloma cells (kindly provided by Sandra McLachlan, Thyroid Autoimmune Disease Unit, Cedars-Sinai Medical Center and University of California Los Angeles) grown in 10% FCS gold IMEM-medium, 2 mM L-Glutamine, 100 U/ml Penicillin and 10 µg/ml Streptomycin (all Sigma-Aldrich, Deisenhofen, Germany), quantified by RAST, and used as a positive control and standard. (Standardcurve and reproducibility see supplemental figure S2)

The detection of IgE-anti-TPO with this assay was confirmed in selected sera by western blot (Supplemental figure S3) and/or classical ELISA after removal of IgG via Protein-G-affinity chromatography and of low molecular weight proteins via ultrafiltration (Supplemental figure 1c).

### Assessment of urticaria activity and serum markers for inflammation, autoimmunity or allergy

Disease activity in all csU patients was determined by use of the UAS7, the urticaria activity score of seven consecutive days (44, 45). Briefly, patients recorded the number of wheals (no wheals  = 0 points, up to 20 wheals = 1 point, 21 to 50 wheals or large confluent wheals = 2 points, and >50 wheals  = 3 points) and the severity of pruritus (no pruritus  = 0 points, mild pruritus  = 1 point, moderate pruritus  = 2 points, severe pruritus  = 3 points) every day during the week before collecting blood for IgE-anti-TPO analyses (range of UAS7 = 0 points to 42 points).

Blood collected from csU patients for IgE-anti-TPO analyses was also investigated for parameters of inflammation (erythrocyte sedimentation rate, blood count, c-reactive peptide, serum electrophoresis), autoimmunity (immunoglobuline levels, thyroid autoantibodies and hormones, complement, circulating immune complexes, rheumatoid factor, indirect immunoflourescent testing, anti-nuclear antibodies, anti-neutrophil antibodies) and allergy (IgE serum levels). These analyses were done by the Charité central laboratory using standard assays.

### Statistics

Descriptive analysis presents means and standard deviations, for the right skewed IgE-anti-TPO measurements medians and the interquartile range (IQR) was calculated.To achieve normal distributions for model based analyses, log transformation was applied if necessary. Correlations were calculated using Spearmańs Rank Test. The diagnostic accuracy for the distinction of csU patients and healthy controls based on their serum levels of IgE-anti-TPO was analysed by the calculation of a ROC curve including selected pairs of sensitivity and specificity, of the area under the curve (AUROC), and of the confidence limits for this curve. In addition, a density estimator for the distribution of IgE-anti-TPO values in the healthy subjects and csU patients was given. CsU patient subgroups with a normal distribution were identified by the method of mixed distribution [28] and characterized by the calculation of theoretical ROC curves. The theoretical ROC curves are obtained by replacing observed values of sensitivity and specificity by the theoretical values obtained from both normal distributions.

## Supporting Information

Figure S1 Differences of a classic ELISA vs. site-directed IgE capture ELISA. Direct ELISA (Suppl. Fig. S1b) was classical performed in Nunc Maxisorp 96 well plates. In brief the wells were loaded with hu rec. TPO 1 µg/ml (RSR-Biochemicals Ltd. Cardiff, UK) in a pH 9,2 100 mM Na-HCO3/Na2HPO4 buffer, followed by blocking with 2% BSA in PBS. After washings diluted serum (1∶25) or standard TPO as calibration standard was applied for 2 hours. Bound IgE was ditected via Fc-biotinylated goat anti huIgE (Sigma-Aldrich, Deisenhofen, Germany, 1∶1000 in PBS) and Streptavidin-horse radish peroxidase (Sigma-Aldrich, 1∶4000 in PBS). Enzymatic stain reaction was then started with 0,02% hydrogen peroxide and 0,02% ABTS (2,2̀-azinodi-(3-ethylbenzthiazoline)-sulfonic acid) in 20 mM Na-citrate buffer at pH 5,0 and stopped with 1% SDS in PBS after 30 min reaction time. The reaction product was measured at 405 nm in an Ascent Multiscan ELISA-plate reader (Thermolab Systems Oy, Turku Finland). This classical ELISA which are usually applicable for detection of IgE towards external antigens failed in detecting auto-IgE in relevant CU sera, although the patients exhibited thyroid pathology and elevated total IgE [*]. After removing the possible competing auto-IgG anti TPO we were able to detect IgE anti-TPO-autoantibody in the same CU sera in classic sandwich ELISA as expected [16] (Extinctions see Fig. S1c). Since large-scaled purification procedures of patient's sera prior to routine ELISA is uneconomic and has difficulties with the reproducibility, we established a special site-directed hu-IgE capture ELISA (Fig. S1b) as described in Materials and Methods. Supplemental Figure S1c: Comparison of an classic ELISA after IgG depletion vs. site-directed IgE capture ELISA Immunospecific detection (optical density OD 405) of IgE-anti-TPO in a defined CU patient's serum by direct ELISA and after Protein-G & anti-IgE affinity chromatography in comparison with site-directed IgE capture ELISA. As control served standard anti-TPO-hIgE measured via direct ELISA (direct ELISA, grey bar). Classical direct ELISA (Fig. S1b) which is usually applicable for detection of IgE towards external antigens failed in detecting auto-IgE-anti-TPO probably due to competing auto-IgG-anti-TPO. When possible competing auto-IgG was removed via Protein-G affinity chromatography and via ultrafiltration through a MW 10000 membrane, this IgE fraction (direct ELISA, black bar) yielded in a much better detectable signal in OD405 in classic sandwich ELISA compared to non purified samples (direct ELISA, white bar). In contrast, the site-directed IgE capture ELISA (Fig. S1a), allows a highly sensitive detection of occurring auto-IgE-anti-huTPO with the same specificity as for purified IgE samples (site-directed IgE capture ELISA, white bar). [*] Concha LB, Chang CC, Szema AM, Dattwyler RJ, Carlson HE (2004) IgE antithyroid antibodies in patients with Hashimoto's disease and chronic urticaria. Allergy Asthma Proc 25: 293–296.
(0.50 MB TIF)

Figure S2 Standardcurve and Reproducibility of the site-directed IgE capture ELISA. The site-directed IgE capture ELISA allows a highly sensitive, straight forward and reproducible detection of auto-IgE-anti-huTPO in the sera of patients. The site-directed IgE capture ELISA showes an almost linear correlation of the standard IgE-anti-TPO with the extinction at 405 nm (S2a). The reproducibility of 10 consecutive measurements of one CU patient with a high IgE-anti-TPO level resulted in a coefficient of variation of 0,127. (S2b)
(1.04 MB TIF)

Figure S3 Immunoblot of purified IgE Fractions of a CU-Patient (A) and a healthy control (B) on microsomal thyroid extrakts run on SDS-PAGE + WB. Proteinstaining of microsomal thyroid extracts (C) and of purified corunning recombinant hTPO (D). Proteins of microsomal thyroid extracts (40 µg TPO/ml) and purified corunning recombinant hTPO were separated on discontinuous SDS polyacrylamide gels (conc. 3%/8% acc.). Electrophoresis was run in a Hoefer SE-260 Mighty VE-chamber (Pharmacia GmbH, Freiburg) at 8°C, 40 mA, for 150 minutes. Western blotting of separated proteins on 0,45 µm nitrocellulose sheets (Schleicher & Schüll, Dassel, Germany) was performed in a Hoefer Mini-Transfer chamber (Pharmacia GmbH, Freiburg, Germany). Afterwards the sheets were cut in strips. One strip with microsomal thyroid extracts (C) and one with recombinant TPO (D) underwent an immediate staining with 0,01% Amidoblack in 10% Acetic acid, 20% MeOH, 70% water. The remaining strip with microsomal thyroid extracts were blocked with 5% milk powder in 150 mM NaCl, 10 mM Tris/HCl pH 8,0, 0,05% Tween 20 (TBST) overnight at 4°C and afterwards incubated for 2 hours in separate bags with purified anti-TPO IgE (diluted 1∶10 in TBST, 1%BSA) of sera taken from a CU patient (A) and health control (B). Specific human IgE antibodies were marked by goat anti-human IgE alkaline phosphatase conjugates (1∶400 in TBST, 1%BSA) for 2 h at 25°C. Dye reaction was started with 50 ml 0,02% Nitro blue tetrazolium in 150 mM Tris/HCl pH 9,6, 100 µl 2 M MgCl2 and 20 µl 0,2% 5-Bromo-4-chloro-3-indolylphoshate (BCIP). After 60 min. incubation time at 25°C the reaction was stopped with water.
(0.45 MB TIF)

## References

[1] Greaves MW (2003). Chronic idiopathic urticaria.. Curr Opin Allergy Clin Immunol.

[2] Mlynek A, Maurer M, Zalewska A (2008). Update on chronic urticaria: focusing on mechanisms.. Curr Opin Allergy Clin Immunol.

[3] Staubach P, Vonend A, Burow G, Metz M, Magerl M (2008). Patients with chronic urticaria exhibit increased rates of
sensitisation to Candida albicans, but not to common moulds.. Mycoses.

[4] Zuberbier T, Asero R, Bindslev-Jensen C, Walter Canonica G, Church MK (2009). EAACI/GA(2)LEN/EDF/WAO guideline: definition, classification and
diagnosis of urticaria.. Allergy.

[5] Levy Y, Segal N, Weintrob N, Danon YL (2003). Chronic urticaria: association with thyroid
autoimmunity.. Arch Dis Child.

[6] Leznoff A, Josse RG, Denburg J, Dolovich J (1983). Association of chronic urticaria and angioedema with thyroid
autoimmunity.. Arch Dermatol.

[7] Zauli D, Deleonardi G, Foderaro S, Grassi A, Bortolotti R (2001). Thyroid autoimmunity in chronic urticaria.. Allergy Asthma Proc.

[8] Grattan CE, Francis DM, Hide M, Greaves MW (1991). Detection of circulating histamine releasing autoantibodies with
functional properties of anti-IgE in chronic urticaria.. Clin Exp Allergy.

[9] Fiebiger E, Maurer D, Holub H, Reininger B, Hartmann G (1995). Serum IgG autoantibodies directed against the alpha chain of Fc
epsilon RI: a selective marker and pathogenetic factor for a distinct subset of
chronic urticaria patients?. J Clin Invest.

[10] Sabroe RA, Fiebiger E, Francis DM, Maurer D, Seed PT (2002). Classification of anti-FcepsilonRI and anti-IgE autoantibodies in
chronic idiopathic urticaria and correlation with disease
severity.. J Allergy Clin Immunol.

[11] Grattan CE (2004). Autoimmune urticaria.. Immunol Allergy Clin North Am.

[12] Sabroe RA, Grattan CE, Francis DM, Barr RM, Kobza Black A (1999). The autologous serum skin test: a screening test for autoantibodies in
chronic idiopathic urticaria.. Br J Dermatol.

[13] Staubach P, Onnen K, Vonend A, Metz M, Siebenhaar F (2006). Autologous whole blood injections to patients with chronic urticaria
and a positive autologous serum skin test: a placebo-controlled
trial.. Dermatology.

[14] Bar-Sela S, Reshef T, Mekori YA (1999). IgE antithyroid microsomal antibodies in a patient with chronic
urticaria.. J Allergy Clin Immunol.

[15] Tedeschi A, Lorini M, Asero R (2001). Anti-thyroid peroxidase IgE in patients with chronic
urticaria.. J Allergy Clin Immunol.

[16] Kadooka Y, Idota T, Gunji H, Shimatani M, Kawakami H (2000). A method for measuring specific IgE in sera by direct ELISA without
interference by IgG competition or IgG autoantibodies to IgE.. Int Arch Allergy Immunol.

[17] Valenta R, Duchene M, Pettenburger K, Sillaber C, Valent P (1991). Identification of profilin as a novel pollen allergen; IgE
autoreactivity in sensitized individuals.. Science.

[18] Appenzeller U, Meyer C, Menz G, Blaser K, Crameri R (1999). IgE-mediated reactions to autoantigens in allergic
diseases.. Int Arch Allergy Immunol.

[19] Dimson OG, Giudice GJ, Fu CL, Van den Bergh F, Warren SJ (2003). Identification of a potential effector function for IgE autoantibodies
in the organ-specific autoimmune disease bullous pemphigoid.. J Invest Dermatol.

[20] Fairley JA, Fu CL, Giudice GJ (2005). Mapping the binding sites of anti-BP180 immunoglobulin E
autoantibodies in bullous pemphigoid.. J Invest Dermatol.

[21] Dopp R, Schmidt E, Chimanovitch I, Leverkus M, Brocker EB (2000). IgG4 and IgE are the major immunoglobulins targeting the NC16A domain
of BP180 in Bullous pemphigoid: serum levels of these immunoglobulins reflect
disease activity.. J Am Acad Dermatol.

[22] Natter S, Seiberler S, Hufnagl P, Binder BR, Hirschl AM (1998). Isolation of cDNA clones coding for IgE autoantigens with serum IgE
from atopic dermatitis patients.. Faseb J.

[23] Valenta R, Natter S, Seiberler S, Wichlas S, Maurer D (1998). Molecular characterization of an autoallergen, Hom s 1, identified by
serum IgE from atopic dermatitis patients.. J Invest Dermatol.

[24] Bunder R, Mittermann I, Herz U, Focke M, Wegmann M (2004). Induction of autoallergy with an environmental allergen mimicking a
self protein in a murine model of experimental allergic asthma.. J Allergy Clin Immunol.

[25] Haapala AM, Hyoty H, Parkkonen P, Mustonen J, Soppi E (1997). Antibody reactivity against thyroid peroxidase and myeloperoxidase in
autoimmune thyroiditis and systemic vasculitis.. Scand J Immunol.

[26] Palma-Carlos AG, Palma-Carlos ML (2005). Chronic urticaria and thyroid auto-immunity.. Allerg Immunol (Paris).

[27] Gangemi S, Saitta S, Lombardo G, Patafi M, Benvenga S (2009). Serum thyroid autoantibodies in patients with idiopathic either acute
or chronic urticaria.. J Endocrinol Invest.

[28] Bohning D, Dietz E, Schlattmann P (1998). Recent developments in computer-assisted analysis of
mixtures.. Biometrics.

