# Supplementary figures and images for: IgE Mediated Autoallergy against Thyroid Peroxidase – A Novel Pathomechanism of Chronic Spontaneous Urticaria?
Source: PLoS One. 2011 Apr 12;6(4):e14794. doi: 10.1371/journal.pone.0014794 (PMC3075251; doi:10.1371/journal.pone.0014794)

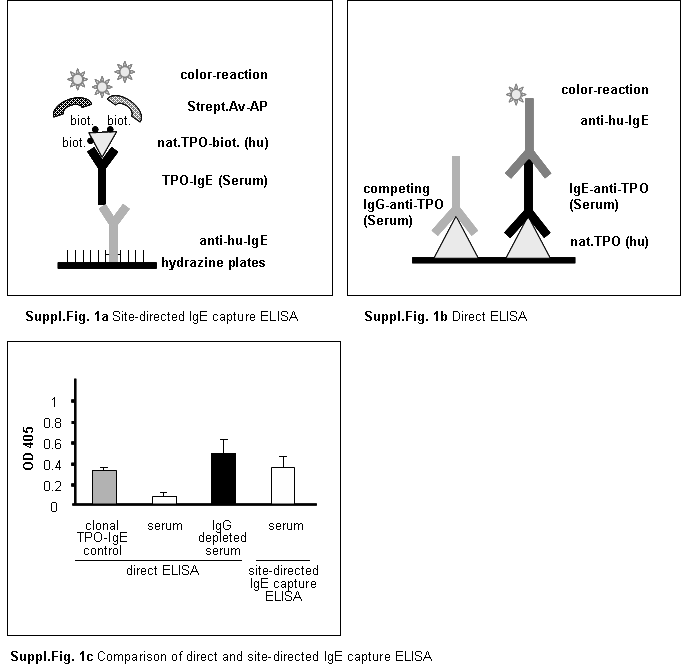

Supplement: Figure S1 — Differences of a classic ELISA vs. site-directed IgE capture ELISA. Direct ELISA (Suppl. Fig. S1b) was classical performed in Nunc Maxisorp 96 well plates. In brief the wells were loaded with hu rec. TPO 1 µg/ml (RSR-Biochemicals Ltd. Cardiff, UK) in a pH 9,2 100 mM Na-HCO3/Na2HPO4 buffer, followed by blocking with 2% BSA in PBS. After washings diluted serum (1∶25) or standard TPO as calibration standard was applied for 2 hours. Bound IgE was ditected via Fc-biotinylated goat anti huIgE (Sigma-Aldrich, Deisenhofen, Germany, 1∶1000 in PBS) and Streptavidin-horse radish peroxidase (Sigma-Aldrich, 1∶4000 in PBS). Enzymatic stain reaction was then started with 0,02% hydrogen peroxide and 0,02% ABTS (2,2̀-azinodi-(3-ethylbenzthiazoline)-sulfonic acid) in 20 mM Na-citrate buffer at pH 5,0 and stopped with 1% SDS in PBS after 30 min reaction time. The reaction product was measured at 405 nm in an Ascent Multiscan ELISA-plate reader (Thermolab Systems Oy, Turku Finland). This classical ELISA which are usually applicable for detection of IgE towards external antigens failed in detecting auto-IgE in relevant CU sera, although the patients exhibited thyroid pathology and elevated total IgE [*]. After removing the possible competing auto-IgG anti TPO we were able to detect IgE anti-TPO-autoantibody in the same CU sera in classic sandwich ELISA as expected [16] (Extinctions see Fig. S1c). Since large-scaled purification procedures of patient's sera prior to routine ELISA is uneconomic and has difficulties with the reproducibility, we established a special site-directed hu-IgE capture ELISA (Fig. S1b) as described in Materials and Methods. Supplemental Figure S1c: Comparison of an classic ELISA after IgG depletion vs. site-directed IgE capture ELISA Immunospecific detection (optical density OD 405) of IgE-anti-TPO in a defined CU patient's serum by direct ELISA and after Protein-G & anti-IgE affinity chromatography in comparison with site-directed IgE capture ELISA. As control se [file pone.0014794.s001.tif]

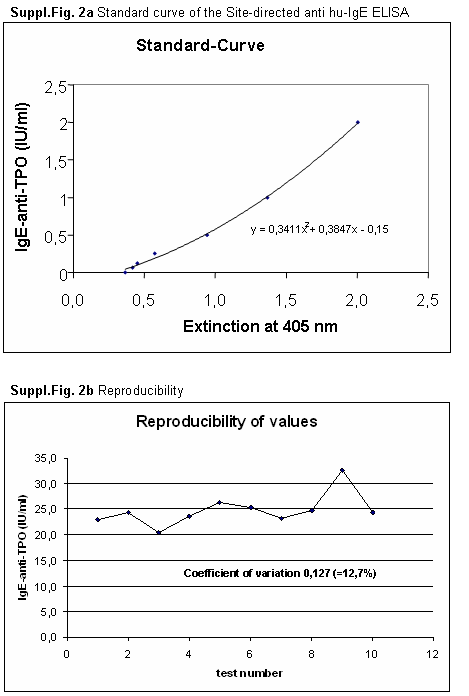

Supplement: Figure S2 — Standardcurve and Reproducibility of the site-directed IgE capture ELISA. The site-directed IgE capture ELISA allows a highly sensitive, straight forward and reproducible detection of auto-IgE-anti-huTPO in the sera of patients. The site-directed IgE capture ELISA showes an almost linear correlation of the standard IgE-anti-TPO with the extinction at 405 nm (S2a). The reproducibility of 10 consecutive measurements of one CU patient with a high IgE-anti-TPO level resulted in a coefficient of variation of 0,127. (S2b) (1.04 MB TIF) [file pone.0014794.s002.tif]

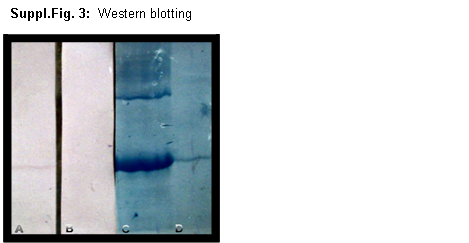

Supplement: Figure S3 — Immunoblot of purified IgE Fractions of a CU-Patient (A) and a healthy control (B) on microsomal thyroid extrakts run on SDS-PAGE + WB. Proteinstaining of microsomal thyroid extracts (C) and of purified corunning recombinant hTPO (D). Proteins of microsomal thyroid extracts (40 µg TPO/ml) and purified corunning recombinant hTPO were separated on discontinuous SDS polyacrylamide gels (conc. 3%/8% acc.). Electrophoresis was run in a Hoefer SE-260 Mighty VE-chamber (Pharmacia GmbH, Freiburg) at 8°C, 40 mA, for 150 minutes. Western blotting of separated proteins on 0,45 µm nitrocellulose sheets (Schleicher & Schüll, Dassel, Germany) was performed in a Hoefer Mini-Transfer chamber (Pharmacia GmbH, Freiburg, Germany). Afterwards the sheets were cut in strips. One strip with microsomal thyroid extracts (C) and one with recombinant TPO (D) underwent an immediate staining with 0,01% Amidoblack in 10% Acetic acid, 20% MeOH, 70% water. The remaining strip with microsomal thyroid extracts were blocked with 5% milk powder in 150 mM NaCl, 10 mM Tris/HCl pH 8,0, 0,05% Tween 20 (TBST) overnight at 4°C and afterwards incubated for 2 hours in separate bags with purified anti-TPO IgE (diluted 1∶10 in TBST, 1%BSA) of sera taken from a CU patient (A) and health control (B). Specific human IgE antibodies were marked by goat anti-human IgE alkaline phosphatase conjugates (1∶400 in TBST, 1%BSA) for 2 h at 25°C. Dye reaction was started with 50 ml 0,02% Nitro blue tetrazolium in 150 mM Tris/HCl pH 9,6, 100 µl 2 M MgCl2 and 20 µl 0,2% 5-Bromo-4-chloro-3-indolylphoshate (BCIP). After 60 min. incubation time at 25°C the reaction was stopped with water. (0.45 MB TIF) [file pone.0014794.s003.tif]
